# Gestational diabetes mellitus: Major risk factors and pregnancy-related outcomes: A cohort study

**DOI:** 10.18502/ijrm.v19i9.9715

**Published:** 2021-10-10

**Authors:** Azam Kouhkan, Laily Najafi, Mojtaba Malek, Hamid Reza Baradaran, Roya Hosseini, Alireza Khajavi, Mohammad Ebrahim Khamseh

**Affiliations:** ^1^Reproductive Epidemiology Research Center, Royan Institute for Reproductive Biomedicine, ACECR, Tehran, Iran.; ^2^Department of Stem Cells and Developmental Biology, Cell Science Research Center, Royan Institute for Stem Cell Biology and Technology, ACECR, Tehran, Iran.; ^3^Endocrine Research Center, Institute of Endocrinology and Metabolism, Iran University of Medical Sciences (IUMS), Tehran, Iran.; ^4^Research Center for Prevention of Cardiovascular Disease, Institute of Endocrinology and Metabolism, Iran University of Medical Sciences (IUMS), Tehran, Iran.; ^5^Department of Andrology, Reproductive Biomedicine Research Center, Royan Institute for Reproductive Biomedicine, ACECR, Tehran, Iran.; ^6^Student Research Committee, Faculty of Paramedical Sciences, Shahid Beheshti University of Medical Sciences, Tehran, Iran.

**Keywords:** Gestational diabetes mellitus, Pregnancy outcomes, Risk factors.

## Abstract

**Background:**

Gestational diabetes mellitus (GDM) is a major pregnancy endocrine problem that has several confirmed risk factors and is associated with adverse pregnancy-related outcomes (PRO).

**Objective:**

To evaluate the relationship between GDM diagnosis and the associated risk factors of PRO (maternal, intrapartum, perinatal, and neonatal) in accordance with International Association of Diabetes and Pregnancy Study Groups criteria.

**Materials and Methods:**

This prospective cohort study was performed with 531 singleton parturient (265 GDM and 266 non-GDM). They were selected consecutively from referral hospitals, and the maternal, intrapartum, perinatal, and neonatal outcomes were assessed.

**Results:**

The major risk factors influencing the GDM diagnosis were maternal age, obesity, family history of diabetes, previous history of GDM, and previous history of macrosomia. In the comparison of PRO between the groups, significant associations were detected for emergency cesarean delivery, preeclampsia, polyhydramnios, premature rupture of membrane, preterm delivery, and neonatal hyperbilirubinemia in the GDM group. In the multivariate logistic regression analysis, a previous history of stillbirth was significantly associated with maternal and perinatal outcomes. The odds ratios (CI 95%) of the PRO in the women with a GDM diagnosis were: maternal = 2.43 (1.51-3.90), intrapartum = 2.05 (1.35-3.11), perinatal = 2.00 (1.29-3.10), and neonatal = 1.68 (1.08-2.62). The PRO was significantly correlated with GDM diagnosis, but not with the risk factors.

**Conclusion:**

The adverse pregnancy outcomes were significantly correlated with GDM diagnosis, and the outcomes were not directly affected by the risk factors. Given the related adverse outcomes for mothers and offspring, early screening and management of GDM is necessary especially in Asians and in low-/middle-income countries.

## 1. Introduction

Chronic diseases such as diabetes have become one of the major public health problems in recent years. One of the main forms of diabetes is gestational diabetes mellitus (GDM), which is recognized as glucose intolerance, and is diagnosed initially during pregnancy. It could affect between 1.3% and 18.6% of pregnancies in Iran (1), depending on the studied population and the diagnostic criteria used.

The pregnancies complicated by GDM are associated with feto-maternal sequelae. The adverse pregnancy-related outcomes (PRO) are spontaneous abortions, macrosomia, intrauterine growth restriction (IUGR), premature rupture of membranes (PROM), neonatal hypoglycemia, respiratory distress, and the need for neonatal intensive care unit (NICU) admission (2). Maternal poor glycemic control is associated with a high prevalence of adverse perinatal outcomes (3).

The most common risk factors for GDM diagnosis are higher age and body mass index (BMI), previous history of GDM, first-degree relatives with diabetes, and adverse obstetric outcomes (4). Parturients diagnosed with GDM are at an increased risk of obesity, metabolic syndrome, and type-II diabetes mellitus for themselves in the future and their offspring in later life (5). Due to the high incidence of metabolic syndrome and genetic predisposition among Asians, they are more likely to have GDM. Therefore, with an increase in GDM globally, identifying major risk factors and adverse feto-maternal outcomes and providing appropriate care to women developing GDM could substantially impact the health of large numbers of parturients and offspring.

The screening criteria, ideal timing for screening, risk factors, and feto-maternal complications of GDM remain under debate. Considering the importance of early detection and appropriate GDM diagnosis, the International Association of Diabetes and Pregnancy Study Groups (IADPSG) (6) has identified new diagnostic criteria, which distinguish and help manage GDM and the adverse outcomes to prevent further complications.

The current study was undertaken to evaluate the relationship between GDM diagnosis and the associated risk factors of PRO in accordance with the IADPSG criteria.

## 2. Materials and Methods

### Subjects

This prospective cohort study was carried out between April 2015 and July 2017. The present study was performed on 531 single women, who were selected consecutively from referral hospitals (Kamali, Akbarabadi and Arash); comprising of GDM and non-GDM pregnancies. The sample size was calculated using the G power software (version 3.1), using power = 90%, α = 5%, β = 0.1, d = 0.05, and the prevalence of GDM = 3.41% and 4.9% (1, 7). Considering the probable drop rate of 5%, the sample size calculated each group was 265 parturient. This sample size was confirmed by the prevalence of feto-maternal outcomes (5.1% preterm in Iran (8), 5% large-for-gestational age [GA] (9), 7.9% meconium-stained amniotic fluid (10) and 8% preeclampsia (11)). The study population was comprised of 265 GDM and 266 non-GDM parturient.

The exclusion criteria were parturients who were transferred out at any GA, smoking or substance abuse, abortion, multifetal pregnancy, gross fetal anomalies, overt diabetes, chronic hypertension, systemic disorders, and use of systemic medications.

A complete history was taken and a physical evaluation was performed for all parturients by a trained physician. Maternal baseline demographic characteristics, clinical and obstetrical parameters, laboratory data and anthropometric variables were obtained from existing antenatal records and face-to-face interviews which were conducted in the first and following prenatal visits by trained observers. For height and weight measurement, a calibrated digital scale (Seca gmbh & co. kg., Germany) was used. The BMI measured in the first trimester, was the best predictor of prepregnancy BMI; it is considered by dividing the weight (kilograms) by the square of the height (meters) (12). Standard measurement of blood pressure (BP) was recorded for all the parturients; defined as a seated position, relax after at least 5 min, refrain from talking or moving and cease smoking and drinking tea or coffee, or eating food for at least half an hr.

GDM is diagnosed at 24-28 wk of gestation using a “one-step strategy” (75-gr 2-hr oral glucose tolerance test) which is well-defined according to the American Diabetes Association/IADPSG criteria (6, 13, 14). The test should be done within 8-14 hr of overnight fasting. Based on the aforementioned guidelines criteria, a diagnosis of GDM can be made when one of the following values is met or exceeded in the one-step strategy: 0-hr (fasting) ≥ 92 mg/dL; 1-hr ≥ 180 mg/dL; or 2-hr ≥ 153 mg/dL.

The non-GDM group was defined as those with a normal oral glucose tolerance test. The enzymatic calorimeter method was used for blood glucose measurement by means of a standard kit (EliTech kit, France). The major risk factors which influenced the GDM diagnosis were defined as follows: maternal age > 35 yr, obesity (BMI ≥ 30 kg/m${}^{2}$), family history of diabetes, previous history of GDM, and previous history of macrosomia (neonate weight ≥ 4000 gr) (14).

### PRO

The following maternal outcomes were included: preeclampsia (as BP ≥ 140/90 mmHg and a positive proteinuria [at least 1+ dipstick, 30 mg/dl] shown by random urine sample or ≥ 300 mg/24 hr, or a urine protein-to-creatinine ratio of ≥ 0.3 after 20 wk of pregnancy (15)), oligohydramnios (< 5${}^{\mathrm{th}}$ percentile of amniotic fluid volume expected for GA), and polyhydramnios (amniotic fluid index > 24 cm expected for GA in the amniotic sac).

The following intrapartum outcomes were included: emergency cesarean section (CS), PROM (rupture of the membrane more than 1 hr before the onset of labor), and preterm delivery (delivery before 37 wk of gestation).

Perinatal outcomes were defined as fetal death, macrosomia (birth weight at delivery > 4,000 gr), IUGR (growth < the third percentile for GA), first- and fifth-min Apgar score > 7, and congenital malformation.

Neonatal outcomes were defined as NICU admission, neonatal hypoglycemia (16), neonatal hyperbilirubinemia (17), and neonatal respiratory distress (18). Neonatal variables were examined by a pediatrician in all cases after delivery.

### Ethical considerations

The ethics committee of Iran University of Medical Sciences, Tehran, Iran approved the study protocol (Code: IR.IUMS.REC.1393.24991) and a written informed consent was signed by all parturients and their spouses included in the study. All procedures performed were in accordance with the ethical standards of institute of endocrinology and metabolism, Iran University of Medical Sciences, Tehran, Iran and with the 1964 Helsinki Declaration and its later amendments.

### Statistical analysis 

The discrete and continuous variables are reported using the number (percent) and mean (standard deviation [SD]), respectively. To compare the continuous variables between GDM and non-GDM groups, *t* test and Mann-Whitney test were used, depending on the variables' distribution is in accordance with the normal distribution or not, respectively. In addition, the Chi-square test was the tool for comparing the categorical variables between GDM and non-GDM groups. Finally, the responses which owned significant levels between two groups were fitted in the ordinal logistic regression model. Besides, the variables of clinical important but non-significant in group-comparison step were also put in the model.

The analyses were performed using the statistical software Statistical Package for the Social Sciences (SPSS) (version 16, SPSS Inc., Chicago, IL, USA) Stata (ver. 12). The significance level was chosen to be 0.05.

## 3. Results

A total of 531 parturients including 265 GDM and 266 non-GDM parturients were included in this study.

The average gravidity in non-GDM and GDM groups was 1.86 ± 0.93 and 2.20 ± 1.00, respectively (p = 0.0001). The parity average in the non-GDM and GDM groups was 0.66 ± 0.78 and 0.90 ± 0.81, respectively (p = 0.0005). The demographic and reproductive characteristics of the parturients are summarized in Table I.

The relationship between GDM and the major risk factors which influenced the GDM diagnosis (maternal age > 35 yr, obesity [BMI ≥ 30 kg/m${}^{2}$], family history of diabetes, previous history of GDM, and previous history of macrosomia [neonate weight ≥ 4000 gr]), were compared between the two groups, using the Chi-square test. The analysis showed significant association between GDM and maternal age > 35 yr (p = 0.0001), obesity [BMI ≥ 30 kg/m${}^{2}$] (p = 0.03), family history of diabetes (p = 0.0001), previous history of GDM (p = 0.0001), and previous history of macrosomia (p = 0.01). Finally, at multivariate logistic regression analysis was used to evaluate the effect of these five risk factors (adjusted for maternal characteristics) on the odds of GDM development (dependent variable), and the same results as above was obtained, with few alterations (findings are not shown).

Table II presents the comparison of the PRO (maternal, intrapartum, perinatal, and neonatal) between the two groups and the association of the aforementioned outcomes with the GDM group.

This study, measured the effect of major risk factors on the PRO (maternal, intrapartum, perinatal, and neonatal) in the GDM group; the results are presented in table III.

Table IV shows the results from the multivariate logistic regression analysis, which was performed on the risk factors for maternal, intrapartum, perinatal, and neonatal outcomes in the study population. The results demonstrated that a previous history of stillbirth was significantly associated with maternal and perinatal outcomes (Table V). The maternal outcomes included preeclampsia, oligohydramnios and polyhydramnios; the intrapartum outcomes were emergency CS, PROM and preterm delivery; the perinatal outcomes were fetal death, macrosomia, IUGR, Apgar 1, 5 and congenital malformation; and the neonatal outcomes were NICU admission, neonatal hypoglycemia, neonatal hyperbilirubinemia, and neonatal respiratory distress.

In an additional analysis, the four aforementioned outcomes were significantly correlated with GDM diagnosis; as the ORs (95% CI, p-value) demonstrate: maternal = 2.43 (1.51-3.90, 0.0001), intrapartum = 2.05 (1.35-3.11, 0.001), perinatal = 2.00 (1.29-3.10, 0.002), and neonatal = 1.68 (1.08-2.62, 0.02).

**Table 1:** Demographic and reproductive characteristics of the parturients


**Variables**	**Non-GDM**	**GDM **	**p-value**
Maternal age (yr)*	28.42 ± 5.26	31.33 ± 5.41	0.0001${}^{a}$
Multigravida${}^{**}$	149 (55.8)	191 (71.8)	0.0001${}^{b}$
Nulliparity${}^{**}$	135 (50.6)	90 (33.8)	0.0001${}^{b}$
Pre- pregnancy BMI (kg/m${}^{2}$)${}^{*}$	24.72 ± 4.37	26.45 ± 4.43	0.0001${}^{a}$
Gestational age at delivery time (wk)${}^{*}$	38.85 ± 1.30	38.07 ± 1.55	0.0001${}^{a}$
Systolic BP (mmHg)${}^{*}$	108.07 ± 11.21	111.97 ± 13.16	0.0003${}^{a}$
Diastolic BP (mmHg)${}^{*}$	70.10 ± 9.23	70.83 ± 9.47	0.3700${}^{a}$
${}^{*}$Data presented as Mean ± SD, ${}^{**}$Data presented as n (%). P-values < 0.05 were considered significant. ${}^{a}$ *t* test, ${}^{b}$Chi-square test. GDM: Gestational diabetes mellitus, BMI: Body mass index, BP: Blood pressure			

**Table 2:** The comparison of pregnancy-related outcomes in GDM and non-GDM groups


**Variables**	Non-GDM*	GDM*	**Odds ratio (95% CI)**	**p-value**
**Emergency CS**	34 (13.03)	58 (22.05)	1.88 (1.18-3.00)	0.007${}^{a}$
**Preeclampsia**	11 (4.170	36 (13.69)	3.64 (1.81-7.33)	0.001${}^{a}$
**Oligohydramnios**	9 (3.47)	7 (2.65)	0.75 (0.27-2.06)	0.58
**Polyhydramnios**	12 (4.63)	28 (10.61)	2.44 (1.21-4.91)	0.01${}^{a}$
**PROM**	6 (2.26)	25 (9.47)	4.53 (1.82-11.24)	0.0001${}^{a}$
**Fetal death**	2 (0.76)	2 (0.75)	0.99 (0.13-7.09)	0.99
**Preterm delivery**	12 (5.11)	29 (11.93)	2.52 (1.25-5.06)	0.008${}^{a}$
**Macrosomia **	7 (2.67)	15 (5.68)	2.19 (0.87-5.47)	0.08
**IUGR**	8 (3.08)	16 (6.06)	2.03 (0.85-4.83)	0.10
**Apgar score at 1 min < 7**	5 (1.87)	9 (3.38)	0.98 (0.56-1.73)	0.27
**Apgar score at 5 min < 7**	0 (0)	2 (0.75)	0.77 (0.42-1.42)	0.15
**Respiratory distress **	28 (10.69)	24 (9.13)	0.83 (0.47-1.49)	0.54
**NICU admission**	10 (6.21)	17 (11.18)	1.90 (0.84-4.29)	0.11
**Neonatal hypoglycemia**	5 (1.89)	7 (2.65)	1.41 (0.44-4.52)	0.55
**Neonatal hyperbilirubinemia**	25 (9.51)	46 (17.42)	2.00 (1.19-3.38)	0.008${}^{a}$
**Congenital malformation**	24 (9.02)	37 (14.02)	1.64 (0.95-2.83)	0.07
*Data presented as n (%), Chi-square test was used.${}^{a}$P-value of 0.05 was considered significant. GDM: Gestational diabetes mellitus, CS: Cesarean section, PROM: Premature rupture of membranes, IUGR: Intra-uterine growth restriction, NICU: Neonatal intensive care unit				

**Table 3:** The effect of major risk factors on PRO (maternal, intrapartum, perinatal, and neonatal) in the GDM group


**Variables**	**Age**	**FHDM**	**BMI**	**PHGDM **	**PHMAC**
**Emergency C/S**	0.73 (0.38-1.41)	1.32 (0.73-2.38)	0.68 (0.29-1.55)	0.38 (0.13-1.13)	0.87 (0.23-3.21)
**Preeclampsia**	0.93 (0.55-1.58)	1.02 (0.62-1.67)	1.26 (0.66-2.37)	1.09 (0.54-2.18)	1.32 (0.46-3.75)
**Oligohydramnios**	0.90 (0.17-4.74)	0.24 (0.02-2.06)	- -	2.89 (0.32-25.70)
**Polyhydramnios**	0.72 (0.29-1.79)	1.59 (0.72-3.49)	1.97 (0.81-4.81)	2.20 (0.86-5.61)	2.24 (0.59-8.47)
**PROM**	1.30 (0.55-3.08)	1.73 (0.75-3.95)	0.62 (0.17-2.18)	0.79 (0.22-2.79)	1.51 (0.32-7.11)
**Fetal death**	- -	- 5.94 (0.36-97.12)	-
**Preterm delivery**	1.69 (0.76-3.75)	1.94 (0.89-4.25)	0.53 (0.15-1.84)	0.94 (0.31-2.90)	1.69 (0.35-8.22)
**Macrosomia **	0.54 (0.15-1.99)	1.01 (0.34-2.92)	0.74 (0.16-3.44)	1.52 (0.41-5.69)	2.79 (0.56-13.69)
**IUGR**	1.38 (0.48-3.94)	1.19 (0.42-3.30)	1.14 (0.30-4.21)	0.84 (0.18-3.85)	1.11 (0.13-9.05)
**Apgar score at 1 min < 7**	1.11 (0.27-4.53)	0.76 (0.19-3.11)	0.56 (0.07-4.61)	0.72 (0.09-5.93)	-
**Respiratory distress **	1.13 (0.46-2.77)	1.10 (0.46-2.58)	0.89 (0.29-2.74)	0.51 (0.11-2.27)	0.69 (0.08-5.55)
**NICU admission**	2.67 (0.96-7.42)	1.05 (0.37-2.92)	0.64 (0.13-3.00)	1.39 (0.36-5.33)	-
**Neonatal hypoglycemia**	- 2.05 (0.45-9.39)	0.90 (0.10-7.92)	0.99 (0.11-8.46)	2.89 (0.32-25.70)
**Neonatal hyperbilirubinemia**	0.86 (0.43-1.75)	0.96 (0.50-1.85)	0.49 (0.18-1.34)	0.87 (0.34-2.22)	0.32 (0.04-2.52)
**Congenital malformation**	1.45 (0.70-2.99)	1.73 (0.86-3.47)	1.56 (0.68-3.58)	1.17 (0.45-3.05)	0.94 (0.20-4.34)
Data presented as the OR (95% CI). *A p-value of 0.05 was considered significant. FHDM: Family history of diabetes, BMI: Body mass index, PHGDM: Previous history of GDM, PHMAC: Previous history of macrosomia, C/S: Cesarean section, PROM: Premature rupture of membranes, IUGR: Intra-uterine growth restriction, NICU: Neonatal intensive care unit					

**Table 4:** The effect of major risk factors on PRO (maternal, intrapartum, perinatal, and neonatal) in the GDM group


**Variables**	**Age**	**FHDM**	**BMI**	**PHGDM**	**PHMAC**
**Emergency CS**	0.73 (0.38-1.41)	1.32 (0.73-2.38)	0.68 (0.29-1.55)	0.38 (0.13-1.13)	0.87 (0.23-3.21)
**Preeclampsia**	0.93 (0.55-1.58)	1.02 (0.62-1.67)	1.26 (0.66-2.37)	1.09 (0.54-2.18)	1.32 (0.46-3.75)
**Oligohydramnios**	0.90 (0.17-4.74)	0.24 (0.02-2.06)	- -	2.89 (0.32-25.70)
**Polyhydramnios**	0.72 (0.29-1.79)	1.59 (0.72-3.49)	1.97 (0.81-4.81)	2.20 (0.86-5.61)	2.24 (0.59-8.47)
**PROM**	1.30 (0.55-3.08)	1.73 (0.75-3.95)	0.62 (0.17-2.18)	0.79 (0.22-2.79)	1.51 (0.32-7.11)
**Fetal death**	- -	- 5.94 (0.36-97.12)	-
**Preterm delivery**	1.69 (0.76-3.75)	1.94 (0.89-4.25)	0.53 (0.15-1.84)	0.94 (0.31-2.90)	1.69 (0.35-8.22)
**Macrosomia **	0.54 (0.15-1.99)	1.01 (0.34-2.92)	0.74 (0.16-3.44)	1.52 (0.41-5.69)	2.79 (0.56-13.69)
**IUGR**	1.38 (0.48-3.94)	1.19 (0.42-3.30)	1.14 (0.30-4.21)	0.84 (0.18-3.85)	1.11 (0.13-9.05)
**Apgar score at 1 min < 7**	1.11 (0.27-4.53)	0.76 (0.19-3.11)	0.56 (0.07-4.61)	0.72 (0.09-5.93)	-
**Apgar score at 5 min < 7**	- -	- -	-
**Respiratory distress **	1.13 (0.46-2.77)	1.10 (0.46-2.58)	0.89 (0.29-2.74)	0.51 (0.11-2.27)	0.69 (0.08-5.55)
**NICU admission**	2.67 (0.96-7.42)	1.05 (0.37-2.92)	0.64 (0.13-3.00)	1.39 (0.36-5.33)	-
**Neonatal hypoglycemia**	- 2.05 (0.45-9.39)	0.90 (0.10-7.92)	0.99 (0.11-8.46)	2.89 (0.32-25.70)
**Neonatal hyperbilirubinemia**	0.86 (0.43-1.75)	0.96 (0.50-1.85)	0.49 (0.18-1.34)	0.87 (0.34-2.22)	0.32 (0.04-2.52)
**Congenital malformation**	1.45 (0.70-2.99)	1.73 (0.86-3.47)	1.56 (0.68-3.58)	1.17 (0.45-3.05)	0.94 (0.20-4.34)
Data presented as the OR (95% CI). *P-value of 0.05 was considered significant. GDM: Gestational diabetes mellitus, FHDM: Family history of diabetes, BMI: Body mass index, PHGDM: Previous history of GDM, PHMAC: Previous history of macrosomia, CS: Cesarean section, PROM: Premature rupture of membranes, IUGR: Intra-uterine growth restriction, NICU: Neonatal intensive care unit					

**Table 5:** The multivariate logistic regression analysis for the risk factors on maternal, intrapartum, perinatal, and neonatal outcomes in the study population


[1.455in,lr]**Risk factors** **Outcomes**	**Maternal**	**Intrapartum**	**Perinatal**	**Neonatal**
**Age ≥ 35 yr **	0.95 (0.55-1.64)	0.93 (0.56-1.53)	1.18 (0.71-1.95)	0.89 (0.51-1.56)
**Family history of diabetes **	0.94 (0.57-1.56)	1.39 (0.89-2.17)	1.21 (0.76-1.93)	1.01 (0.62-1.65)
**Obesity (BMI ≥ 30 kg/m${}^{2}$)**	1.25 (0.68-2.29)	0.92 (0.51-1.66)	1.16 (0.64-2.08)	1.02 (0.52-1.98)
**Previous history of GDM**	1.91 (0.94-3.89)	0.52 (0.23-1.21)	1.19 (0.58-2.47)	0.69 (0.28-1.66)
**Previous history of macrosomia (neonate weight ≥ 4000 gr)**	1.31 (0.45-3.80)	1.41 (0.44-4.45)	0.85 (0.27-2.66)	1.16 (0.34-3.96)
**Previous history of abortion**	0.91 (0.52-1.58)	0.85 (0.51-1.41)	1.12 (0.67-1.86)	1.29 (0.76-2.21)
**Previous history of stillbirth**	3.63 (1.16-11.32)*	1.56 (0.49-4.96)	3.65 (1.23-10.79)*	0.93 (0.15-5.74)
Data presented as the odds ratio (95% CI). *P-values < 0.05 were considered significant. BMI: Body mass index, GDM: Gestational diabetes mellitus				

## 4. Discussion 

The aim of the present study was to evaluate the relationship between the GDM diagnosis and the associated risk factors of PRO in accordance with the IADPSG criteria.

The major risk factors that were associated with the GDM diagnosis were maternal age, obesity, family history of diabetes, previous history of GDM, and previous history of macrosomia. In the comparison of PRO between groups, the significant associations were detected for emergency CS delivery, preeclampsia, polyhydramnios, premature rupture of membrane, preterm delivery, and neonatal hyperbilirubinemia in the GDM group. A previous history of stillbirth was significantly associated with maternal and perinatal outcomes. All PRO were significantly correlated with GDM diagnosis, but not with the risk factors.

Early identification of GDM is crucial because it affects clinical decision-making. Optimal GDM management, including lifestyle alterations, medical nutrition therapy, insulin therapy, and antepartum fetal observation, may decrease the perinatal morbidity and mortality associated with GDM (13). However, screening and accurately detecting GDM in asymptomatic pregnant women is controversial. Screening is done using a one-step strategy at least once at or about 24 wk of gestation unless there are suggestions for it to be done earlier (2). Healthcare providers should recognize and screen high-risk groups of pregnant women to detect and manage GDM earlier. Our data showed that major risk factors (such as age, obesity, family history of diabetes, previous history of GDM, and previous history of macrosomia) significantly increased the risk of GDM. In most countries, early screening is performed based on parameters such as belonging to a specific ethnic group related to a high GDM prevalence, advanced maternal age, prepregnancy obesity, history of diabetes in first-degree relatives, previous history of GDM, previous large-for-GA babies, and glucosuria. Early screening using only traditional risk factors may increase the likehood of missing GDM cases (19). Therefore, we suggest that both traditional risk factors and new biomarkers should be researched in large populations and with varied ethnic groups.

In our regional literature review, we detected a varied range of GDM prevalence values from 1.3% to 18.6% in Iranian pregnant women (1). Ethnicity seems to play a principal role as well: the trend of GDM in the Asian population is increasing; also increased insulin resistance is observed at much lower BMI levels in Asian compared with Europeans (20). Although South Asians are a high-risk population, screening, risk factors and complications of GDM are still debated (21). The recognized risk factors (such as age, BMI, family history of diabetes, previous history of macrosomia/congenital malformation, and previous history of GDM) were mainly attained from studies of European populations (22). Furthermore, there was a Malaysian and Iranian study which examined this relationship (2, 23) and found similar results. Keshavarz and colleagues showed that older age, a family history of diabetes, obesity, previous macrosomia, and glycosuria can be risk factors for GDM (23). In another study, the risk of GDM was greater in parturients aged > 35 yr with a fourfold increase that was related to pancreatic β-cell function and insulin-sensitivity falling with age (24). In addition, a 2.45-fold increase of risk has been attributed to obesity (2), which is related to the elevation of insulin resistance that occurs as a result of obesity (19). In the present study, GDM risk was higher by 3.08-fold and 1.7-fold in women > 35 yr and with a BMI ≥ 30 kg/m${}^{2}$, respectively. The placenta and adipose tissue that produce a large number of diabetogenic adipokines. Tumor necrosis factor-alpha, which is a as diabetogenic adipokine, plays an important role in insulin-resistance pathways and may induce adverse feto-maternal outcomes (25). In two studies, a family history of DM was presented in 77.7% and 16.3% of GDM cases (26, 27). Our findings showed that a family history of diabetes increased the risk of GDM by 2.90-fold. Keshavarz and colleagues suggested that screening based on risk factors alone could miss 16% of GDM cases (23). A recent systematic review evaluated the cost-effectiveness of identification and/or treatment of GDM. This study found that neither early screening nor treating GDM seems to be convincingly cost-effective in high-income countries, but they suggested that early screening and proper detection of GDM might be worthwhile in low-/middle- incomes countries due to different health systems and other health priorities (28).

The present study revealed that adverse PRO such as emergency CS, preeclampsia, polyhydramnios, PROM, preterm delivery, and neonatal hyperbilirubinemia were significantly associated with GDM diagnosis (OR = 1.88, 3.64, 2.44, 4.53, 2.52, and 2.00, respectively) compared to non-GDM women. All the PROs were significantly correlated with GDM diagnosis. Other studies have shown that the risk of fetal macrosomia (29) may be increased by a diagnosis of GDM, and a greater potential risk may exist for shoulder dystocia in macrosomic infants (30). Additionally, CS delivery, spontaneous miscarriage, preterm delivery, a low Apgar score, the need for NICU admission, hypoglycemia, congenital malformations and respiratory distress syndrome occured more commonly among the infants of GDM parturients (2). Another Iranian study reported a higher risk of pregnancy complications and adverse feto-maternal outcomes with GDM (23). In GDM parturients, a higher risk of having a non-spontaneous vaginal delivery such as operative deliveries was observed in GDM paturients compared with non-GDM women (OR = 1.9) (2). GDM is not only associated with short-term adverse feto-maternal outcomes, such as macrosomia, increased CS rates, hypertensive disorders, and fetal hyperinsulinemia (31), but also significantly increases the risk for long-term adverse events for both mothers and their offspring (19).

Nilofer and co-workers and Opara and colleagues found that hyperbilirubinemia is a common adverse event in GDM, which complicated 20% and 57.4% of the infants they studied, respectively; this resulted from excessive red blood cell breakdown in association with polycythemia because of immature bilirubin conjugation by the liver of the neonate (26, 32). In contrast to our findings, Logakodie and colleagues revealed no significant difference in the fetal outcomes of GDM parturients, which may be due to the tight glycemic control of GDM status (2). In the present study, the major risk factors were not associated with PRO. According to our multivariate logistic regression analysis, after group adjustment, a previous history of stillbirth was an independent risk factor for maternal and perinatal outcomes. Unlike in our study, Jolly and colleagues who observed that maternal obesity was associated with adverse pregnancy outcomes; however, they found similar results to our study in relation to the association of GDM with adverse pregnancy outcomes (24).

## 5. Limitations

In the present study, we could not evaluate the socioeconomic status or dietary patterns of the parturients.

## 6. Conclusion

In conclusion, we found that the PRO were significantly correlated with GDM diagnosis, but not with the risk factors. Given the increased rate of GDM worldwide, healthcare providers should attend to risk factors for early diagnosis of GDM. In view of the long- and short-term adverse related outcomes for mothers and offspring, early screening and appropriate management of GDM are necessary, especially in Asians and in low-/middle-income countries. Detection of predictive factors of GDM should be researched in these populations.

##  Conflict of Interest 

The authors declare that there is no conflict of interest that could be perceived as prejudicing the impartiality of the research reported.
